# Simultaneous GC-FID Quantification of Main Components of *Rosmarinus officinalis* L. and *Lavandula dentata* Essential Oils in Polymeric Nanocapsules for Antioxidant Application

**DOI:** 10.1155/2019/2837406

**Published:** 2019-02-10

**Authors:** Perla Giovanna Silva-Flores, Luis Alejandro Pérez-López, Verónica Mayela Rivas-Galindo, David Paniagua-Vega, Sergio Arturo Galindo-Rodríguez, Rocío Álvarez-Román

**Affiliations:** ^1^Departamento de Química Analítica, Facultad de Medicina, Universidad Autónoma de Nuevo León, Av. Fco. I. Madero y Dr. E. Aguirre Pequeño S/N, 64460 Monterrey, Nuevo León, Mexico; ^2^Cátedras CONACYT-UANL, Departamento de Química Analítica, Facultad de Medicina, Universidad Autónoma de Nuevo León, Av. Fco. I. Madero y Dr. E. Aguirre Pequeño S/N, 64460 Monterrey, Nuevo León, Mexico; ^3^Departamento de Química Analítica, Facultad de Ciencias Biológicas, Universidad Autónoma de Nuevo León, Av. Pedro de Alba S/N, 66455 San Nicolás de los Garza, Nuevo León, Mexico

## Abstract

The essential oils (EO) of *R. officinalis* and *L. dentata* have been widely used due to their antioxidant activity. However, due to their high volatility, the loading of EO into polymeric nanocapsules (NC) represents an efficient way of retaining their effect in future topical administration. In this way, the quantitative determination of EO incorporated into NC is necessary for simultaneous monitoring of the main components of the EO during the nanoencapsulation process as well as for precise and exact dosing of the components used during the performance of *in vitro* and *in vivo* biological tests. In this study, EO were isolated by hydrodistillation in a Clevenger-type apparatus and characterized by GC-MS and GC-FID analyses. The major constituents of EO-*R. officinalis* were camphor (39.46%) and 1,8-cineole (14.63%), and for EO-*L. dentata* were 1,8-cineole (68.59%) and *β*-pinene (11.53%). A new analytical method based on GC-FID for quantification of free and encapsulated EO was developed and validated according to ICH. Linearity, limit of detection and quantification, and intra- and interday precision parameters were determined. The methods were linear and precise for the quantification of the main components of EO. The EO were encapsulated by nanoprecipitation and were analyzed by the GC-FID method validated for their direct quantification. The NC size was 200 nm with homogeneous size distribution. The quantification of the incorporated EO within a NC is an important step in NC characterization. In this way, an encapsulation efficiency of at least 59.03% and 41.15% of total EO-*R. officinalis* and EO-*L. dentata*, respectively, was obtained. Simple, repeatable, and reproducible methods were developed as an analytical tool for the simultaneous quantification of the main components of EO loaded in polymeric nanocapsules as well as their monitoring in biological assays.

## 1. Introduction

Essential oils (EO) have been used for years in pharmaceuticals, cosmetics, and food products due to their health benefits [1], as antimicrobials [2], antidiabetics [3], analgesics [4], antioxidants [5], anti-inflammatories [6], and sedatives and anxiolytics [7]. EO are complex mixtures of volatile compounds that contain about 20 to 60 components at different concentrations. In general, it has been found that the main components could determine the biological properties of EO in living beings [1, 8]. In this sense, it has been reported that the EO of *Rosmarinus officinalis* (EO-*R. officinalis*) has antioxidant [9, 10], antimicrobial [11], anti-inflammatory, and antinociceptive [12, 13] properties, mainly due to the presence of monoterpenes such as 1,8-cineol, camphor, and *α*-pinene [9]. Similarly, the EO of *Lavandula dentata* (EO-*L. dentata*), belonging to the same family *Lamiaceae*, have been widely used because of their pharmacological effects as an anticonvulsant and antidepressant [14], sedative [15], and antioxidant [16]. These biological activities are attributed to the presence of monoterpenes such as linalool, camphor, and 1,8-cineol [17].

However, EO are highly volatile and can decompose easily due to direct exposure to heat, moisture, light, or oxygen [18]. Nanoencapsulation represents a feasible and efficient alternative to overcome these drawbacks since it would provide stability to the active substance [19], protection against oxidation [20], retention of volatile compounds, and reduction of side effects [20, 21]. In recent years, there has been growing interest in the development of polymeric nanoparticles, particularly nanocapsules (NC), as systems for the administration of lipophilic bioactive components [22], representing a new form of topical administration of EO with a preventive and/or therapeutic antioxidant effect. In this study, Eudragit EPO was used as the NC-formed polymer due to its cationic nature which will allow a greater interaction with the skin, as a result of the electrostatic interaction [23].

On the other hand, the chemical characterization and the quantitative determination of EO incorporated in NC arise as a need for the simultaneous monitoring of the main components of the EO during the nanoencapsulation process and the precise and exact dosing of the components used during the performance of *in vitro* and *in vivo* biological tests.

In this sense, the quantification of EO by HPLC has been limited by the high volatility and low UV absorptivity that most of these components have, presenting a greater application in the analysis of its nonvolatile components or other natural products (i.e., extracts) [24, 25]. In contrast, gas chromatography with mass spectrometry (GC-MS) has been used, given the ionization properties of the volatile components of the EO; however, it is an analytical technique that presents difficulties in the identification of the signals of these complex samples due to the fact that many terpenes have identical mass spectra as a consequence of the close similarities in fragmentation patterns and rearrangements after ionization, in addition to having a prolonged analysis time and high cost. [26–28].

Gas chromatography with a flame ionization detector (GC-FID) represents an analytical technique suitable for the qualitative and quantitative analysis of EO since it offers high sensitivity, great stability, and an exceptionally high linear dynamic range that allows the analysis of volatile components of the EO at very low concentrations or at trace levels [29, 30]. To date, there is no analytical method validated by GC-FID by direct injection whose applicability is the simultaneous and quick quantification of essential oil components in polymeric NC.

The main objective of this work was to develop and validate an analytical method by GC-FID by direct injection for the simultaneous quantification of the main monoterpenes of free and nanoencapsulated EO of *R. officinalis* and *L. dentata* for future application as an antioxidant agent in biological assays.

## 2. Material and Methods

### 2.1. Plant Materials and Reagents

*R. officinalis* and *L. dentata* were collected from Monterrey, N. L., Mexico. The specimens were identified in the Herbarium of the School of Biological Sciences, Universidad Autónoma de Nuevo Leon, Mexico. Dichloromethane (Baker, USA), methanol (Tedia, USA), acetone (CTR Scientific, USA), and isopropyl alcohol (Chromadex, USA) were of HPLC grade. Purified water was from a Milli-Q water-purification system (Veolia, USA). 1,8-cineol (>98%), *β*-pinene (98.5%), camphor (>95%), 4-allylanisole (98%), and linalool (>95%) were of GC grade (Aldrich, USA). The standard solution was n-alkanes (C8-C24, Sigma-Aldrich, USA). Eudragit EPO, an NC-formed polymer, was obtained from Evonik Industries, Germany.

### 2.2. Essential Oil Extraction

The essential oils were isolated from the aerial parts of fresh plants of *R. officinalis* (EO-*R. officinalis*) and *L. dentata* (EO-*L. dentata*) by hydrodistillation using a Clevenger-type apparatus for 4 h. The essential oils obtained were stored in a sealed vial at −4° C until chromatographic analysis. The yield percentage was calculated as weight (g) of essential oils per 100 g of the plant.

### 2.3. Essential Oil Analysis

#### 2.3.1. Gas Chromatography-Mass Spectrometry (GC-MS)

The composition of volatile constituents of the essential oil was analyzed using a gas chromatograph 6890N (Agilent Technologies, USA) equipped with a 5973 INERT mass selective spectrometer (ionization energy 70 eV) and an HP-5MS column (30 m × 0.25 mm × 0.25 *µ*m). Helium (99.99%) was the carrier gas at a flow rate of 0.5 mL min^−1^. Data acquisition was performed in the scan mode. The ionization source temperature was 230°C, quadrupole temperature was 150°C, and injector temperature was 220°C. The oven temperature was programmed as follows: 35°C for 9 min, increased from 35°C to 150°C at 3°C min^−1^, held at 150°C for 10 min, increased to 250°C at 10°C min^−1^, increased at 3°C min^−1^ to 270°C, and held at 270°C for 10 min. The samples were injected as mentioned above. Components were identified by comparing retention indices relative to C8–C20 n-alkanes, and the mass spectra were compared with the mass spectra from the US National Institute of Standards and Technology (NIST) library and the reference data [31].

#### 2.3.2. Gas Chromatography with Flame Ionization Detection (GC-FID)

The GC-FID analysis of the EO was performed with a gas chromatograph Clarus 480 (Perkin Elmer, USA) equipped with a flame ionization detector and an Elite-5 capillary column (30 m × 0.25 mm × 0.25 *μ*m; PerkinElmer, USA). The oven temperature was programmed as mentioned above. The detector and injector temperatures were 280°C and 220°C, respectively. The carrier gas was helium (99.99%) at a flow rate of 0.5 mL min^−1^. Essential oil samples (2 *μ*L) were injected using the split mode. The percentage composition of EO was calculated using the peak normalization method.

### 2.4. Preparation of Standard Solutions

Standard solutions were prepared by dilution of a stock solution (50 mg·mL^−1^) prepared from the two main components of each essential oil in the following concentrations: EO-*R. officinalis*, 1,8-cineol (20 mg·mL^−1^) and camphor (30 mg·mL^−1^) and EO-*L. dentata*, *β*-pinene (7 mg·mL^−1^) and 1,8-cineol (43 mg·mL^−1^).

The working solutions were prepared in methanol in a concentration working interval using linalool (100 *µ*g·mL^−1^) and 4-allylanisol (100 *µ*g·mL^−1^) as internal standards for the EO-*R. officinalis* and EO-*L. dentata*, respectively. Standards were stored at −4°C and protected from light until use.

### 2.5. Validation Method by GC-FID

The quantitative analysis of the main components used as standard solutions and present in nanocapsules (NC) was performed by GC-FID by direct injection using a gas chromatograph Clarus 480 (Perkin Elmer, USA) equipped with a flame ionization detector and an Elite-5 capillary column (30 m × 0.32 mm i.d. 0.25 *μ*m film thickness; PerkinElmer, USA). For the *R. officinalis* essential oil, the oven temperature was held at 60°C for 1 min, increased at 10°C min^−1^ to 160°C, increased at 20°C min^−1^ to 200°C, and finally increased at 15°C min^−1^ to 230°C (and held) for 1 min. While for the *L. dentata* essential oil, the oven temperature was held at 70°C for 1 min, increased at 8°C min^−1^ to 130°C (and held) for 1 min, increased at 17°C min^−1^ to 170°C, and finally increased at 25°C min^−1^ to 230°C. The detector and injector temperatures were 280°C and 220°C, respectively. The carrier gas was helium (99.99%) at a flow rate of 1.0 mL·min^−1^. The injection volume was 2 *μ*L using the split mode.

### 2.6. Method Validation

The method was validated in terms of linearity, limit of detection (LOD), limit of quantification (LOQ), and accuracy and precision (intra- and interday) according to International Conference on Harmonisation guidelines [32].

The linearity was determined by calculating a regression line from the plot of the peak area versus concentration in a range from 10 to 160 *μ*g·mL^−1^ in triplicates. The linearity was evaluated by calculation of a regression line using the least squares method.

The LOD and LOQ were calculated from the calibration curve according to

(1) $$\begin{array}{c} \text{LOD}=\frac{3.3\sigma }{s}, \end{array}$$

(2) $$\begin{array}{c} \text{LOQ}=\frac{10\sigma }{s}, \end{array}$$

where *σ* is the standard deviation of the response and *s* is the slope of the calibration curve. The residual standard deviation of the line regression or the standard deviation of *y*-intercepts of the line regression may be used as the standard deviation.

Accuracy was determined by calculating the recovery percentage using the standard addition method. The determination of the concentration of 1,8-cineol and camphor in the EO-*R. officinalis* and *β*-pinene and 1,8-cineol in the EO-*L. dentata* was performed by analyzing the NC loaded with EO added with five levels of total concentration of standards (range 10–160 *μ*g·mL^−1^) in triplicates. The average concentration value obtained for each level was compared with the theoretical value, which was considered to be 100%, and the SD was determined.

Precision was evaluated in terms of repeatability (intraday precision) and intermediate precision (interday precision) and was expressed by percentage recovery and the SD for each main component.

Repeatability was determined by analyzing EO-loaded NC added with three levels of concentration (10, 40, and 160 *μ*g·mL^−1^) with six replicates each, during a single day. The intermediate precision was evaluated for repeatability during three different days.

### 2.7. Nanoencapsulation of the *R. officinalis* and *L. dentata* Essential Oils

EO-loaded NC were prepared according to the nanoprecipitation method developed by Fessi et al. [33]. The organic phase was prepared by dissolving 450 mg of Eudragit EPO® and 225 mg of EO-*R. officinalis* or EO-*L. dentata* in 15 mL of organic solvent mixture (acetone:isopropyl alcohol (50 : 50)) under magnetic stirring at room temperature. The organic solution was added to 20 mL of the aqueous phase (Milli-Q water) under moderate magnetic stirring (125 rpm). Finally, the organic solvent mixture was eliminated by dialysis. Briefly, the suspension was transferred to a membrane of regenerated cellulose Spectrum/Por (Spectrum Labs, USA), which was placed in a container with distilled water in agitation at 25°C during 2 hours with two changes of water. NC characterization was carried out to determine mean size, zeta potential, and polydispersity index (PDI) by dynamic light scattering (DLS) (Zetasizer Nano ZS 90, Malvern Instruments, UK) at 25°C. The stability of NC was determined from the particle size during 8 weeks (25 ± 2°C) in terms of means size and the PDI.

### 2.8. Method Applicability

#### 2.8.1. Determination of Encapsulation Loading (% EL) and Encapsulation Efficiency (% EE)

Once the chromatographic methods were development and validated, the amount of EO-*R. officinalis* and *L. dentata* in the NC was determined indirectly. The NC dispersions were centrifuged at 25,000 rpm at 3°C for 2 h (Allegra 64R Centrifuge, Beckman Coulter, USA). The supernatant was removed and the pellet was washed thrice with methanol to ensure the complete solubilization of monoterpenes. Methanol solutions were analyzed by the previously validated GC-FID method in order to quantify the monoterpenes in NC. Finally, the %EL (3) and %EE (4) were derived as follows:

(3) $$\begin{array}{c} \text{EL\%}=\frac{\left(\text{amount of EO added}-\text{amount of EO not encapsulated}\right)}{\left(\text{total polymer}+\text{amount of EO added}\right)}*100\text{,} \end{array}$$

(4) $$\begin{array}{c} \text{EE\%}=\frac{\left(\text{amount of EO added}-\text{amount of EO not encapsulated}\right)}{\left(\text{amount of EO added}\right)}*100\text{.} \end{array}$$

## 3. Results and Discussion

### 3.1. Extraction of the Essential Oils

In the present study, the EO were obtained by hydrodistillation with a yield percentage of 0.72 ± 0.19% (w/w) and 0.53 ± 0.29% (w/w) for *R. officinalis* and *L. dentata*, respectively. The yield of EO as well as their variations according to the origin of the plant has been reported in different investigations [34]. The yield obtained for the *R. officinalis* essential oil was higher than that reported by Bekkara et al., with a yield of 0.8% for the plant from Algeria [35], while the yield of lavender EO was lower than that reported by Imelouane et al., with a yield of 1.41% for *L. dentata* from Morocco [36]. This variability may be due to different collection times and/or climatic conditions at the plant-collection sites [37]. For example, Zaouali et al. affirmed that variations in the yield from different varieties of *Rosmarinus* EO from Tunisia could be attributed to variations in climatic conditions [10].

### 3.2. Chemical Composition of Essential Oils

**Figure 1:**
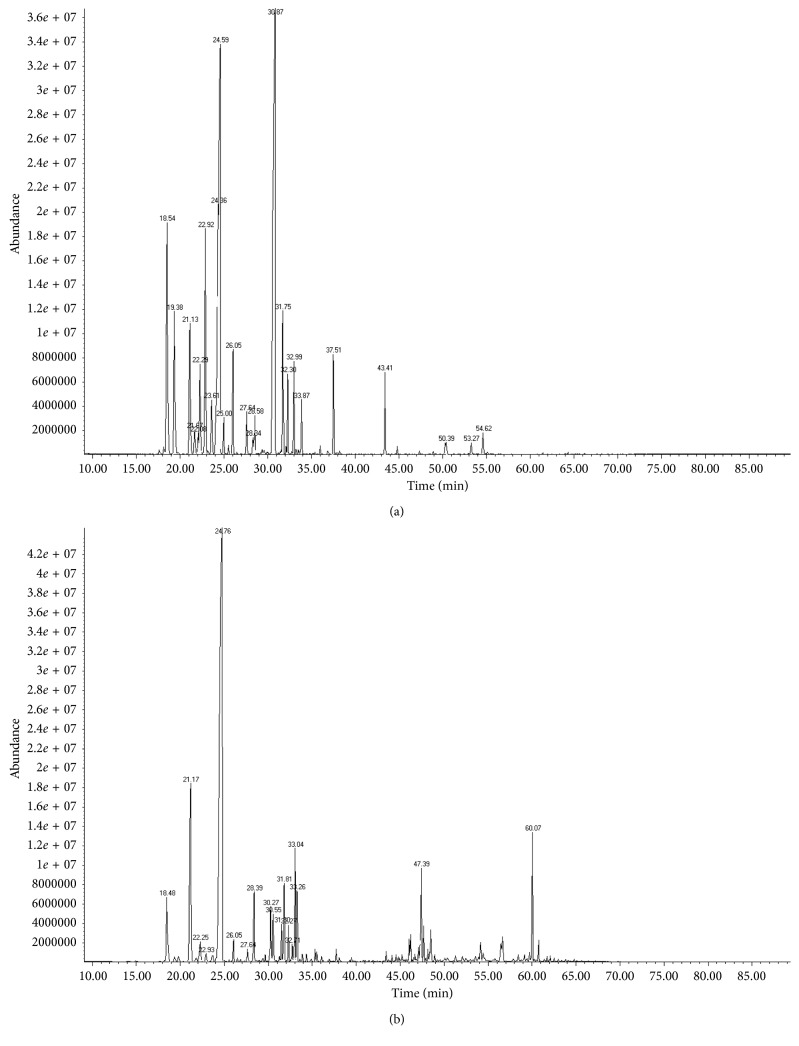
Chromatogram GC-MS of essential oils of *R. officinalis* (a) and *L. dentata* (b).

Essential oils were analyzed by GC-MS and GC-FID to identify their constituents. In Figure 1(a) and 1(b), the GC-MS chromatograms of the EO of *R. officinalis* and *L. dentata*, respectively, are presented.

A total of 22 components of the *R. officinalis* essential oil were identified. The EO-*R. officinalis* composition was characterized by the presence of oxygenated monoterpenes (62.34%) represented mainly by camphor (39.46%) and 1,8-cineole (14.63%) (Figure 1(a)).

In *L. dentata* essential oil, 16 components were identified; the principal constituents were oxygenated monoterpenes (80.71%) and hydrogenated monoterpenes (16.19%); the main constituents were 1,8-cineole (68.59%) and *β*-pinene (11.53%) (Figure 1(b)).

The retention time, retention indices, and chemical composition of essential oils are presented in Table 1.

Rašković et al. identified in *R. officinalis* essential oils 1,8-cineole (43.77%), camphor (12.53%), *α*-pinene (11.51%), *β*-pinene (8.16%), and camphene (4.55%) as the main constituents [38]. A lower concentration of 1,8-cineol (18.7–21.6%) was described by Ojeda-Sana et al. [34]. Touati et al. quantified the main components in the essential oil of *L. dentata* leaves, 1,8-cineole (33.54%) and camphor (18.9%) [39].

The above data agree with the assertion that the composition of the essential oil depends on several factors, such as climate and soil; hence, there may be differences in the percentage of its components.

### 3.3. Method Validation by GC-FID

The validation of the method for the quantification of the two main components in EO was performed to know the linearity, precision, and accuracy of the chromatographic method for the quantification of the peak components of EO-loaded NC. The chromatographic conditions and performance parameters were adjusted to provide a simple analysis with the best peak resolution, reducing run time and lowering the cost of validation and analysis of essential oils.

The regression equation, the correlation coefficient, LOD, and LOQ of each main component used as standards were established and are shown in Table 2. The standard curves of each main component were prepared by plotting the response area versus concentration. The acceptance criterion for linearity is given by the correlation coefficient, which must be greater than or equal to 0.99 [40]. A good linearity response was obtained with the method developed for the two main components of each essential oil used as standards. These values make it possible to ensure that the methods are capable of quantifying the main compounds in the established range.

The results obtained for the LOD and LOQ were between 0.48 and 1.89 *μ*g·mL^−1^ and 0.15 and 3.88 *μ*g·mL^−1^ for EO-*R. officinalis* and EO*-L. dentata*, respectively (Table 2). These results can be compared with those obtained by Fancello et al. for the components of *Citrus* limon var. pompia leaf essential oil. They calculated and obtained the limits of detection and quantification in a concentration range 50 to 150 *µ*g·mL^−1^ and 100 to 300 *µ*g·mL^−1^, respectively [41]. In comparison with these results, the values obtained in this work make it possible to guarantee that the method is capable of detecting trace amounts, either in free or encapsulated essential oils.

Accuracy was determined by analyzing five concentrations of the standard solution using a standard addition technique. A percentage recovery close to 100% indicates high accuracy of the analytical method. Miao et al. established an analytical method for quantitative analysis and chemical fingerprinting of volatile oils from *Alpinia oxyphylla*. They also established that the validated GC-FID method showed a good recovery with recovery percentages close to 100% [42]. In Table 3 are shown the values of percentage recovery and SD for accuracy, precision intermediate, and reproducibility of the main components of essential oils in NC.

### 3.4. Method Applicability

#### 3.4.1. Preparation and Characterization of EO-Loaded Polymeric Nanoparticles

**Figure 2:**
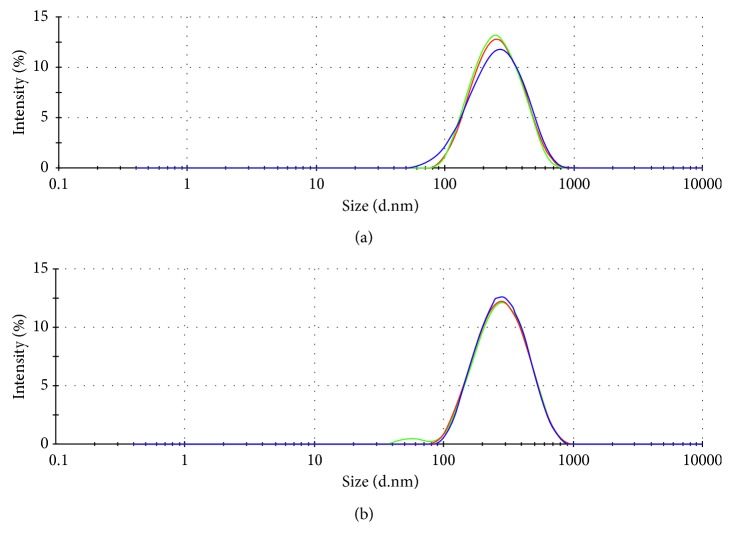
Size distribution of nanoparticles of EO-*R. officinalis* (a) and EO-*L. dentata* (b) measured by the DLS technique (8 weeks at 25 ± 2°C).

The nanoprecipitation technique described by Fessi et al. [33] was used for EO-loaded NC preparation. Previously, it had been established that the ideal size in a biological system having loaded NC is about 200 nm [43]. The size of each EO-loaded NC was greater than 200 nm with homogeneous distribution and stability during 8 weeks at 25 ± 2°C (Figure 2). A percentage of encapsulation loading and efficiency are shown in Table 4. Specifically, a %EE of 59.03% for 1,8-cineol and camphor content in total *R. officinalis* essential oil and 41.15% for *β*-pinene and 1,8-cineol in total *L. dentata* essential oil were obtained, indicating that at least 59.03% and 41.15% of the *R. officinalis* and *L. dentata* essential oils, respectively, were encapsulated during the preparation of the NC. These results were lower than those reported with NC of *Origanum vulgare* ssp and *Thymus capitatus* essential oils (96 ± 4% and 91 ± 1%, respectively) [44]. The results revealed that the *in situ* properties of the NC-polymer determine the %EE of essential oil components. For example, Lugo-Estrada et al. used a headspace solid-phase microextraction GC-FID method to demonstrate that the %EE of *Thymus vulgaris* essential oil in NC of *ε*-polycaprolactone was close to 96% [45].

The results of %EL obtained in the present work may be due to the volatile nature of the EO and were in agreement with the findings on the loading of oregano essential oil in chitosan nanoparticles, which have been reported by Hosseini et al. They developed formulations of chitosan nanoparticles with oregano essential oil with a percentage of encapsulation loading in a range of 1.32 to 2.12% [22]. The hydrophobic characteristics of EO make them a good candidate for encapsulation in nanoparticular systems via nanoprecipitation. However, it is important to emphasize that the encapsulation of a complex natural product, such as EO, is a process that represents greater difficulty compared to the encapsulation of a drug, and it requires studies of optimization of EO encapsulation [46].

Finally, a number a studies employing UV-VIS spectroscopy have been conducted to investigate encapsulation efficiency and loading capacity of EO-NC. A disadvantage of these UV techniques is that they generate an overlap of essential oil components signal, which may obtain an overestimation, and the UV-vis methods used have not been previously validated.

## 4. Conclusions

In this study, the chemical compositions of the essential oils from leaves of *R. officinalis* and *L. dentata* were determined. *β*-pinene, 1,8-cineole, and camphor were identified as the main components of the two essential oils. In this study, the validated analytical method allowed the simultaneous quantification of two main components of each nanoencapsulated essential oil, allowing the direct monitoring of these components in biological tests *in vitro* and *in vivo*. Hence, it is of great importance, due to the lack of analytical methods by direct GC-FID, to analyze these nanoencapsulated essential oils reported in the literature.

## Figures and Tables

**Table 1:** Chemical composition percentage of essential oils (% relative to the peak area).

Composition	RI^a^	RI^b^	*R. officinalis*		*L. dentata*	
			Rt (min)	Peak area (%)	Rt (min)	Peak area (%)
*Monoterpene hydrocarbons*						
*α*-Pinene	934	939	18.54	9.43	18.48	2.87
Camphene	949	954	19.38	4.97	—	—
*β*-Pinene	977	979	21.13	4.75	21.17	11.53
Myrcene	993	990	22.29	3.24	22.26	0.55
*α*-Phellandrene	1003	1002	22.92	6.37	—	—
*α*-Terpinene	1017	1017	23.61	1.11	22.93	1.24
*β*-Phellandrene	1031	1029	24.36	2.30	—	—
(Z)-*β*-ocimene	1043	1050	25.00	0.52	—	—
*γ*-Terpinene	1061	1059	26.05	1.86	—	—
Terpinolene	1088	1088	27.64	0.60	—	—
Total				**35.14**		**16.19**

*Oxygenated monoterpenes*						
1,8-Cineole	1035	1031	24.59	14.63	24.76	68.59
*β*-Linalool	1100	1096	—	—	28.39	1.63
*trans*-Sabinene hydrate	1100	1098	28.58	0.56	—	—
*trans*-Pinocarveol	1139	1139	—	—	30.27	1.15
Camphor	1145	1146	30.87	39.46	30.55	1.03
Pinocarvone	1163	1164	—	—	31.50	0.85
Borneol	1168	1169	31.75	2.79	—	—
*δ*-Terpineol	1169	1166	—	—	31.81	1.83
Terpinen-4-ol	1178	1177	32.30	1.18	32.27	0.57
Cryptone	1186	1185	—	—	32.71	2.94
*α*-Terpineol	1191	1188	32.99	1.56	33.04	1.11
Myrtenol	1196	1195	—	—	33.26	1.01
Verbenone	1209	1205	33.87	0.98	—	—
Bornyl acetate	1287	1285	37.51	1.18	—	—
Total				**62.34**		**80.71**

*Sesquiterpene hydrocarbons*						
E-caryophyllene	1421	1419	43.41	1.07	—	—
Total				**1.07**		—

*Sesquiterpene oxygenated*						
Caryophyllene oxide	1581	1582	50.28	0.61	—	—
Total				**0.61**		—

*Others*						
3-Octanone	990	983	22.08	0.35	—	—
Octen-3-ol	984	979	21.67	0.49	—	—
No identified	—	—	—	—	31.27	0.47
No identified	—	—	—	—	60.75	2.64
Total				**0.84**		**3.11**

RI^a^: retention indices; Rt:^b^: retention time; acalculated; breference.

**Table 2:** Analytical parameters of linearity, LOD, and LOQ of the main components of essential oils in standard solutions.

Essential oil	Main component	Regression equation	Correlation coefficient (*R* ^2^)	LOD (*μ*g mL^−1^)	LOQ (*μ*g mL^−1^)
*R. officinalis*	1,8-Cineol	*y* = 1.213*x* + 0.045	0.999	0.48	1.45
	Camphor	*y* = 1.082*x* + 0.111	0.999	0.62	1.89

*L. dentata*	*β*-Pinene	*y* = 1.437*x* − 0.057	0.999	0.15	0.45
	1,8-Cineol	*y* = 1.226*x* + 0.186	0.999	1.28	3.88

**Table 3:** Analytical parameters of accuracy, precision intermediate, and reproducibility of the main components of essential oils in NC.

Essential oil	Main component	Accuracy (% recovery ± SD)	Repeatability (% recovery ± SD)	Precision intermediate (% recovery ± SD)
*R. officinalis*	1,8-Cineol	100.53 ± 4.46	97.26 ± 4.41	98.80 ± 4.87
	Camphor	99.98 ± 5.03	99.19 ± 3.97	100.36 ± 4.84

*L. dentata*	*β*-Pinene	91.17 ± 5.57	98.02 ± 4.77	98.40 ± 4.97
	1,8-Cineol	100.98 ± 4.90	101.95 ± 5.07	99.31 ± 5.75

Values are expressed as mean ± SD.

**Table 4:** Characterization of EO-loaded polymeric NC.

Essential oil	Size (nm)	PDI	Zeta potential (mV)	Main component	% EL	% EE
*R. officinalis*	226.50 ± 5.46	0.197 ± 0.032	54.47 ± 0.45	1,8-Cineol	2.95 ± 0.14	44.13 ± 3.13
				Camphor	2.41 ± 0.13	14.90 ± 1.20

*L. dentata*	235.62 ± 7.50	0.214 ± 0.022	50.40 ± 0.75	*β*-Pinene	1.56 ± 0.30	28.69 ± 5.42
				1,8-Cineol	3.18 ± 0.44	12.46 ± 1.71

Values are expressed as mean ± SD (*n* = 3).

## Data Availability

Currently, the data obtained have been used in the patent process in Mexico. The patent involves the formation of nanoparticles and the quantification of essential oils and their antioxidant activity for topical application. Therefore, the data cannot be released and they could not be sent until the moment of acceptance of the patent.
